# Application of a magnetically separable Zr-MOF for fast extraction of palladium before its spectrophotometric detection

**DOI:** 10.1186/s13065-024-01171-w

**Published:** 2024-03-30

**Authors:** Amin Piri, Massoud Kaykhaii, Mostafa Khajeh, Ali Reza Oveisi

**Affiliations:** 1 Department of Chemistry, Faculty of Sciences, University of Sistan and Baluchestan, Zahedan, 98135-674 Iran; 2 Department of Chemistry, University of Zabol, P.O. Box: 98615-538, Zabol, Iran

**Keywords:** Palladium, Magnetic metal-organic framework, MIP-202, Spectrophotometry, Wastewater analysis, Experimental design

## Abstract

In this research, a novel magnetic zirconium-based metal-organic framework (Fe_3_O_4_@SiO_2_@MIP-202, MMOF), was fabricated, fully characterized, and applied for the batch-mode solid phase extraction of trace amounts of Pd^2+^ ions from water and wastewater samples before its spectrophotometric detection. Pd^2+^ ions were desorbed from MMOF by nitric acid and were complexed by treating with KI solution to have a maximum absorbance at 410 nm. The synthesized MMOF composite showed a very large surface area (65 m^2^.g^− 1^), good magnetization (1.7 emu.g^− 1^) and a large pore volume (0.059 cm^3^.g^− 1^) with adsorption capacity of 194.5 mg of Pd^2+^ ions/g of the adsorbent. This nanosorbent boasts chemo-mechanical stability, high adsorption capacity due to its vast active sites, and facile recovery facilitated by its magnetic properties. Parameters affecting the extraction efficiency of the method were optimized as pH of the sample 7.4, volume of the sample 25 mL, 15 mg adsorbent, 1 mL of 0.1 M HNO_3_ eluent, with 10 and 15 min as the extraction and desorption times, respectively. The calibration curve was found to be linear across the 10.0–1500.0 µg.L^− 1^ range with a limit of detection of 1.05 µg.L^− 1^. The obtained extraction efficiency and enrichment were 98% and 245, respectively. The total analysis time was less than 30 min. This MMOF has never been used for the extraction of Pd^2+^ ions before.

**Supplementary Information:**

The online version contains supplementary material available at 10.1186/s13065-024-01171-w.

## Introduction

Among all platinum metals, palladium (Pd) has the most varied industrial applications such as in autocatalysis, jewelry making and orthopedic stomatology to make dental prostheses. It is also utilized in many electrical devices as a substitute for gold in galvanic components because of its high stability and excellent conductivity. It is also employed in anti-cancer pharmaceuticals [1]. Pd is applied in the process of creating nitric acid and petroleum in the chemical industry. It’s a key ingredient in making synthetic polymers like nylon and rubber. Pd-containing alloys find value in fuel cell production. However, there are significant drawbacks to utilizing Pd, including its expensive price, its toxicity in greater quantities, and its scarcity as a naturally occurring material [2]. Despite the valuable uses and benefits of Pd, its high use causes it to be released into the atmosphere and contaminate food and water sources, and bioaccumulation of this pollution occurs in living organisms. Entering toxic and carcinogenic Pd in living organisms leads to asthma, allergies, nasal conjunctivitis, etc. Due to the increased use and toxicity of Pd compounds for mammals, fish and higher plants, it is very important to extract and determine Pd^2+^ in the environment. This monitoring can also be lead to the discovery of ore resources [3]. Various methods such as liquid-liquid extraction [4], membrane filtration [5], and ion exchange [6] have been used for the extraction of Pd^2+^ ions. Incomplete extraction, waste release in large quantities, high prices, manpower needs, and reduced selectivity are some of the issues with these approaches.

Solid-phase extraction (SPE) makes it a popular and practical way to recover metal species. It is economical, easy-to-use, more selective, and relies less on organic solvents. Until recently, most examples of adsorbents for SPE of Pd^2+^ have been based on chitosan [7, 8], graphene oxide [9], functionalized mesoporous silica [10], and ionic liquids [11]. However, these adsorbents show a weak interaction with Pd^2+^ions and are difficult to separate and recover from the aqueous medium. On the other hand, the porosity and surface area of these materials are low, so they have low adsorption capacities. Poor mechanical properties and low stability in the water also cause the need for a more suitable adsorbent [12, 13].

Metal-organic frameworks (MOFs) are a new category of porous materials. They are three-dimensional crystalline low-density structures that are made up of inorganic nodes and organic ligands [14]. Due to their ability to be predesigned and synthesized systematically, MOFs may have a range of favorable characteristics, such as a large surface area, high porosity (from Å to nm), high durability, and peculiar characteristics [15]. After being synthesized, a variety of functional groups may be added to the lattice to modify the physicochemical properties of MOFs. MOFs have found a wide range of applications in chemical analysis [16] to extract and preconcentrate various analytes such as dyes [17–19], pesticides [20], organic pollutants [21], and so on. One of the most popular kinds of MOF materials are zirconium MOFs owing to their superior chemical, thermal, and mechanical stability and minimal toxicity [22]. The remarkable stability of them is due to the strong interactions between oxophilic Zr^4+^ sites and the carboxylate linkers. Adsorption performance is enhanced by MOFs owing to their huge accessible surface area and porosity, which allows for facile transport of ions and molecules within the 3D framework. Since then, a variety of MOFs made of Zr have been employed as adsorbents to remove or extract different metals [23, 24]. The vast area of regular MOF architectures and interactions between target ions and Zr nodes drive adsorption performance.

Introducing benzene-1,4-dicarboxylic acid in the parent MOF or modifying MOFs by post-synthetic techniques all significantly affect the efficiency, selectivity and adsorption capacity of a MOF for the removal of metals [25, 26]. For the removal of Cd^2+^ and Pb^2+^, the thiourea-modified UiO-66-NH_2_ demonstrated sorption capacities of 117 and 232 mg.g^− 1^, respectively, which were greater than those of the original MOF [27]. The adsorption capacity of allylsulfanyl-UiO-66 MOF for Pb^2+^ was obtained as 45.4 mg.g^− 1^, which shows the efficient extraction of this ion [28]. Ethylenediaminetetraacetic acid acid was also employed as a post-synthetic chelating group on MOF-808, and the sorbent showed an affinity for 22 metal ions, comprising soft ones like Pd and hard ones like Cd [29]. Lin et al. (2018) showed that the selectivity of UiO-66-NH_2_ towards Pd^2+^ ions is approximately 180 times greater than that towards Pt^4+^. This enhanced selectivity was attributed to the stronger binding affinity of the protonated amino groups towards PdCl_4_^2−^ and the better diffusion of PdCl_4_^2−^ through the UiO-66-NH_2_’s pores [30]. In another report, a ZrCl_4_-based MOF was prepared by grafting 2,6-aminopyridine on 3-formyl-4-hydroxybenzoic acid, which was synthesized and used for effective adoption of Pd^2+^ from water. The adsorption capacity was 191.27 mg Pd^2+^/g after 3 h of equilibrium time. The experimental results show that the adsorption of Pd^2+^ ions on MOF is a monolayer and endothermic chemisorption [31].

In in order to improve Pd^2+^ ions adsorption and increase extraction efficiency, as well as prepare an adsorbent that can be easily removed from the extraction phase, in this research work, a magnetic adsorbent based on zirconium nanocomposite (Fe_3_O_4_@SiO_2_@-MIP-202), was prepared and used for Pd^2+^ extraction. To the best of our knowledge, this adsorbent has never been used previously for the adsorption and extraction of metallic cations. MIP-202 (MIP: Materials of the Institute of Porous Materials from Paris) is one of the bio-based Zr-MOF materials derived from amino acids (L-aspartate), exhibiting hydrolytic and chemical stability. This MOF is environmentally friendly and can be easily scalable up to a few grams. Integration of MIP-202 with magnetic silica nanoparticles, can enhance both MOF stability and available sites for capturing palladium ions. The magnetic character of Fe_3_O_4_@SiO_2_@MIP-202 stands as another crucial factor, facilitating its easy retrieval from the extraction environment.

## Experimental

### Materials

All reagents and solvents were of analytical reagent grade and obtained from Sigma-Aldrich Chemical Company (St. Louis, Missouri, US) and utilized unmodified. To prepare an aqueous stock solution of Pd^2+^ with a concentration of 1 g.L^− 1^, 0.01 g of PdCl_2_ salt was dissolved in a 10 mL flask containing some deionized water and filled to the mark line with deionized water. The prepared solution was stored in a refrigerator. KI stock solution (1 g.L^− 1^) was also prepared in deionized water by dissolving solid KI in deionized water. Afterwards, a suitable concentration of KI was prepared and utilized as a ligand for Pd^2+^ complexation.

### Apparatus

A double beam UV/VIS spectrophotometer (Beijing Beifen-Ruili Analytical Instrument Co. Ltd., model UV-2100 (Beijing, China)) was applied to record the absorption spectra. The analytical wavelength of the Pd-iodide complex was found to be 410 nm. Fourier transform infra-red (FTIR) spectra were obtained between 500 and 4000 cm^− 1^ employing a Spectrum 400 FTIR (PerkinElmer, USA). After synthesis, the Fe_3_O_4_@SiO_2_@MIP-202 MOF was characterized by means of the following instruments. A scanning electron microscope (SEM) (TES-CAN, Czech Republic) model EDX MIRA3 was employed to take images. Powdered X-ray diffraction (PXRD) patterns in the range of 1.5°<2θ < 50° were obtained utilizing a powder diffractometer (Philips X’pert, The Netherlands) under Cu K radiation (λ = 1.5418 Å, 293 K). Differential thermal analysis (DTA)/thermogravimetric analysis (TG) were performed by using a Hitachi Instrument, Inc., Tokyo (Japan) model STA7200RV Thermal Analyzer. Samples were heated from 298 to 973 K at a heating rate of 373 K.min^− 1^. A Micromeritics TriStar II 3020 (USA) porosity and surface area analyzer with TriStar II 3020 V1.03 software was utilized to measure the N_2_ adsorption-desorption isotherms at 77 K. pH of the solution were measured employing a Swiss-made pH meter (model 630 Metrohm) with a glass electrode. At least three independent replicates of each experiment were conducted and the average results were recorded. Magnetic properties of the nanocomposite were measured by using a vibrating-sample magnetometer (VSM) Quantum Design, Germany model 8607 with a 7-inch magnet at room temperature. The Brunauer–Emmett–Teller (BET) method was used to measure the surface area of nanocomposite with a Micromeritics Gemini 2360 surface analyzer (USA).

### Synthesis and characterization of Fe_3_O_4_@SiO_2_@-MIP-202 MMOF

Synthesis of the MMOF was performed in three steps. At first, Fe_3_O_4_ magnetic nano-particles (MNPs) were synthesized and used as the magnetic core of the MMOF. In the next step, from this MNPs, Fe_3_O_4_@SiO_2_ microspheres were made which at the final step were used in a reaction for the synthesis of the final adsorbent.

### Preparation of the nanoparticles and nanocomposite

#### Synthesis of Fe_3_O_4_ magnetic nano particles

Fe_3_O_4_ magnetic particles were synthesized according to solvothermal process [32]. Briefly, 7.20 g sodium acetate and 2.7 g FeCl_3_ were dissolve in 100 mL ethylene glycol by rapid stirring. After homogenization, the yellow solution was placed in a Teflon-coated autoclave and heated to 473 K for 8 h. The autoclave was then cooled to normal temperature and resultant black magnetite particles were dried in a vacuum for 12 h at 333 K after several ethanol washing. From SEM images, the size of the particles was found to be between 200 and 300 nm.

#### Synthesis of Fe_3_O_4_@SiO_2_ microspheres

SiO_2_ as a core shell was coated on the MNPs by the following procedure. 0.10 g of Fe_3_O_4_ MNPs were treated with 50 mL of 0.1 M aqueous solution of HCl for 10 min in an ultrasonic with a power of 400 W. MNPs were poured into a solution of the mixture of 80 mL EtOH, 20 mL DI water, and 1.0 mL of 28% wt (concentrated) NH_4_OH aqueous solution. In the next step, tetraethyl orthosilicate (0.03 g, 0.144 mmol) was added to the dispersion and stirred at 298 K for 6 h. By using a magnet, Fe_3_O_4_@SiO_2_ microspheres were separated from the solution, rinsed with EtOH, and water and dried under vacuum at 333 K for 6 h [33].

#### Synthesis of MMOF

**Fig. 1:**
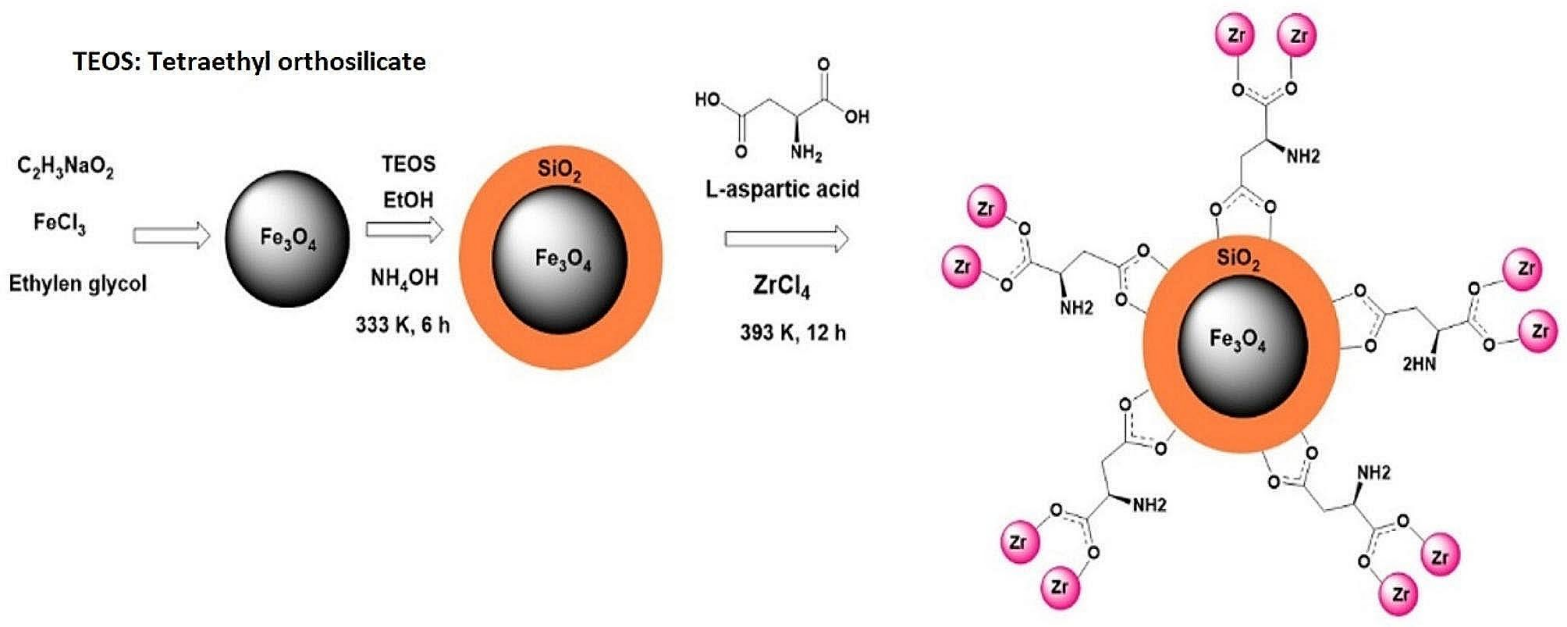
Schematic diagram of the preparation of Fe_3_O_4_@SiO_2_@MIP-202

Ultrasonic was utilized to disperse 0.25 g of Fe_3_O_4_@SiO_2_ into 10 mL of water for 1 h. ZrCl_4_ (1.15 g; 4.93 mmol) and L-aspartate (1.4 g; 10.52 mmol) were added to it and 10 mL of water added. The solution was then heated at 393 K in an oven for 12 h. The vial was cooled to room temperature and the resultant solid was separated by employing an external magnet, and rinsed several times with ethanol. Then, the solid phase was immersed in 80 mL of ethanol for 12 h. Using an external magnet, MMOF powder was collected and dried for one day in a 298 K vacuum oven. A schematic of the preparation of the Fe_3_O_4_@SiO_2_@-MIP-202 MMOF is presented in Fig. 1.

### SPE procedure by using MMOF

All SPE experiments were conducted at ambient temperature. 25.0 mL of the sample solution was placed in a glass test tube and its pH was adjusted to 7.4 by the addition of either 0.1 M HNO_3_ or 0.1 M NaOH. Then, 0.015 g of homogenous MMOF adsorbent was added and stirred for 10 min. After extraction, under an external magnetic field, MMOF was kept inside the tube and the solution was discarded. Then the adsorbent was washed with water several times. Pd^2+^ was desorbed from the MMOF by addition of 1.0 mL of 0.1 M HNO_3_ [34]. Examination of different concentrations of HNO_3_, HCl, and H_2_SO_4_ acids revealed that the highest efficiency was obtained when 0.1 M HNO_3_ is applied. Following desorption, the extracted Pd^2+^ was complexed with a 20% (w/v) KI solution, forming a pale orange complex. While the reaction of Pd (II) with iodide produces a precipitate of palladium iodide, in the large iodide excess of iodide, the soluble palladium complex of PdI_4_^2−^ is formed. For quantitative analysis, the complex was transferred to a spectrophotometer and measured at 410 nm.

## Results and discussion

### Characterization of the Fe_3_O_4_@SiO_2_@MIP-202 MOF

**Fig. 2:**
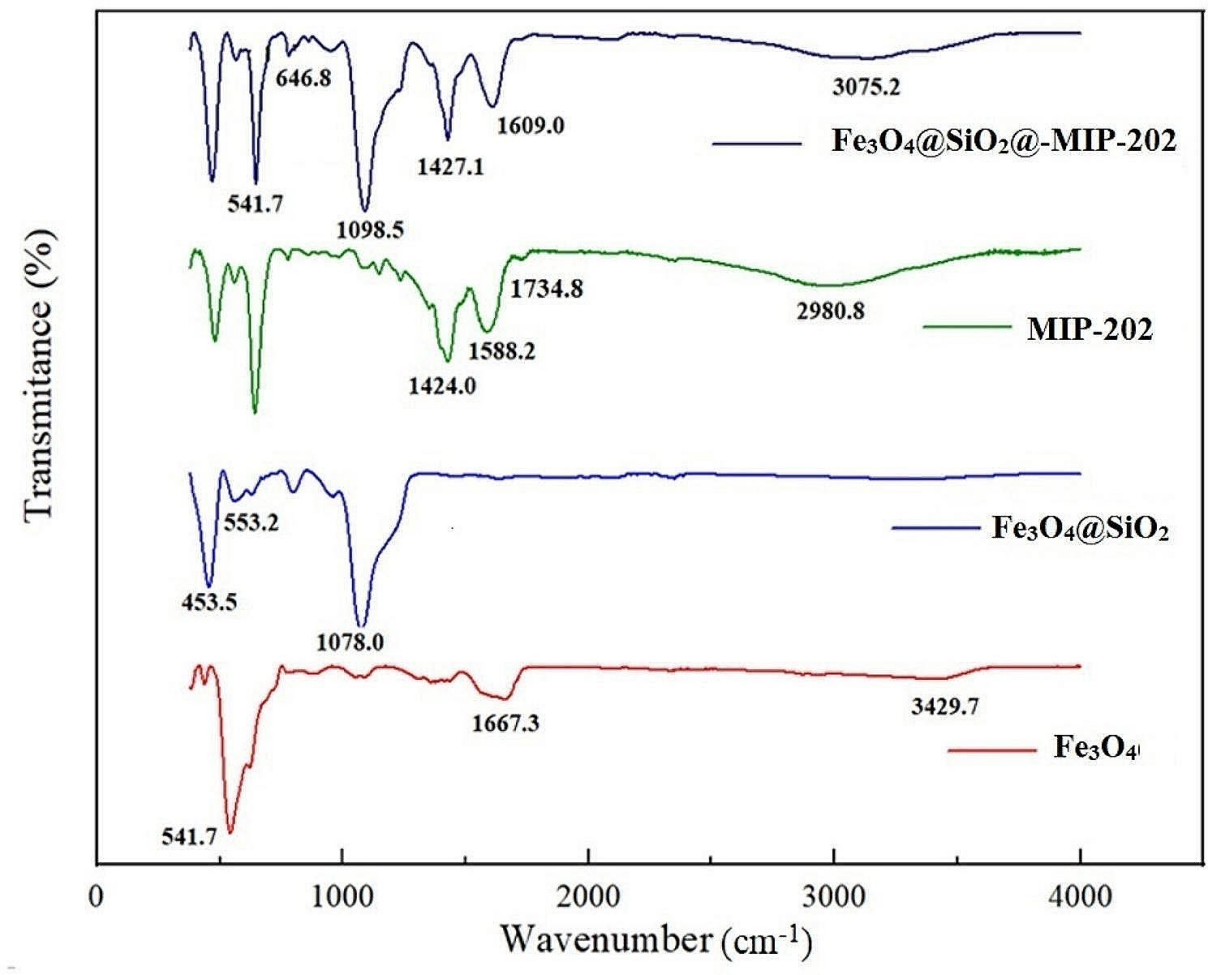
FT-IR spectra of Fe_3_O_4_, Fe_3_O_4_@SiO_2_, MIP202 and Fe_3_O_4_@SiO_2_@MIP-202 nanocomposite

Several methods were used to characterize the MMOF nanoparticles. The FTIR spectra of Fe_3_O_4_, Fe_3_O_4_@SiO_2_, MIP-202 and MIP-202@Fe_3_O_4_@SiO_2_ are presented in Fig. 2. Two peaks of 1667.3 cm^− 1^ and 3429.7 cm^− 1^ at the spectrum of Fe_3_O_4_ can be assigned as the stretching and bending vibrations of the O-H bonds in water, respectively [35]. Since Fe_3_O_4_ nanoparticles were synthesized in water, their surface hydroxyl groups (OH^–^) are abundant [36]. The dominant phase of the prepared particles is magnetite, which is confirmed by the absorption band appearing around 541.7 cm^− 1^ (vibrational and torsional modes of Fe-O bonds) [37]. There is a peak at 453.5 cm^− 1^ which indicates the bending vibration of the Si-O appears at 553.2 cm^− 1^ and 1078.0 cm^− 1^ (Si-O), indicating the successful formation of Fe_3_O_4_@SiO_2_ nanocomposite [38]. The FT-IR spectrum of MIP-202 contains C = O stretching for the carboxyl groups of amino acids at 1734.8 cm^− 1^ [39]. Also, two peaks were appeared in the low-frequency domain, at 1588.2 cm^− 1^ and 1424.0 cm^− 1^ for the NH_2_ bending vibration and CN stretching, respectively. The N-H stretching vibrations are characterized by peaks about 3070 cm^− 1^ which is ovelapped with O-H bands of Fe_3_O_4_@SiO_2_@-MIP-202 [36]. These shifts in peak positions between the pure MIP-202 and its composite with Fe_3_O_4_ and SiO_2_ suggest changes in the chemical environment of the amine groups due to the presence of the metal oxide and silica, which can affect the electronic structure and hydrogen bonding interactions of the NH groups [40]. The peak at 541.7 cm^–1^ is due to the bending vibration of the Si-O bond, while the 6468 cm^–1^ peak is the Fe-O absorption band. As a result, the FT-IR spectrum shows the successful synthesis of MIP-202.

**Fig. 3:**
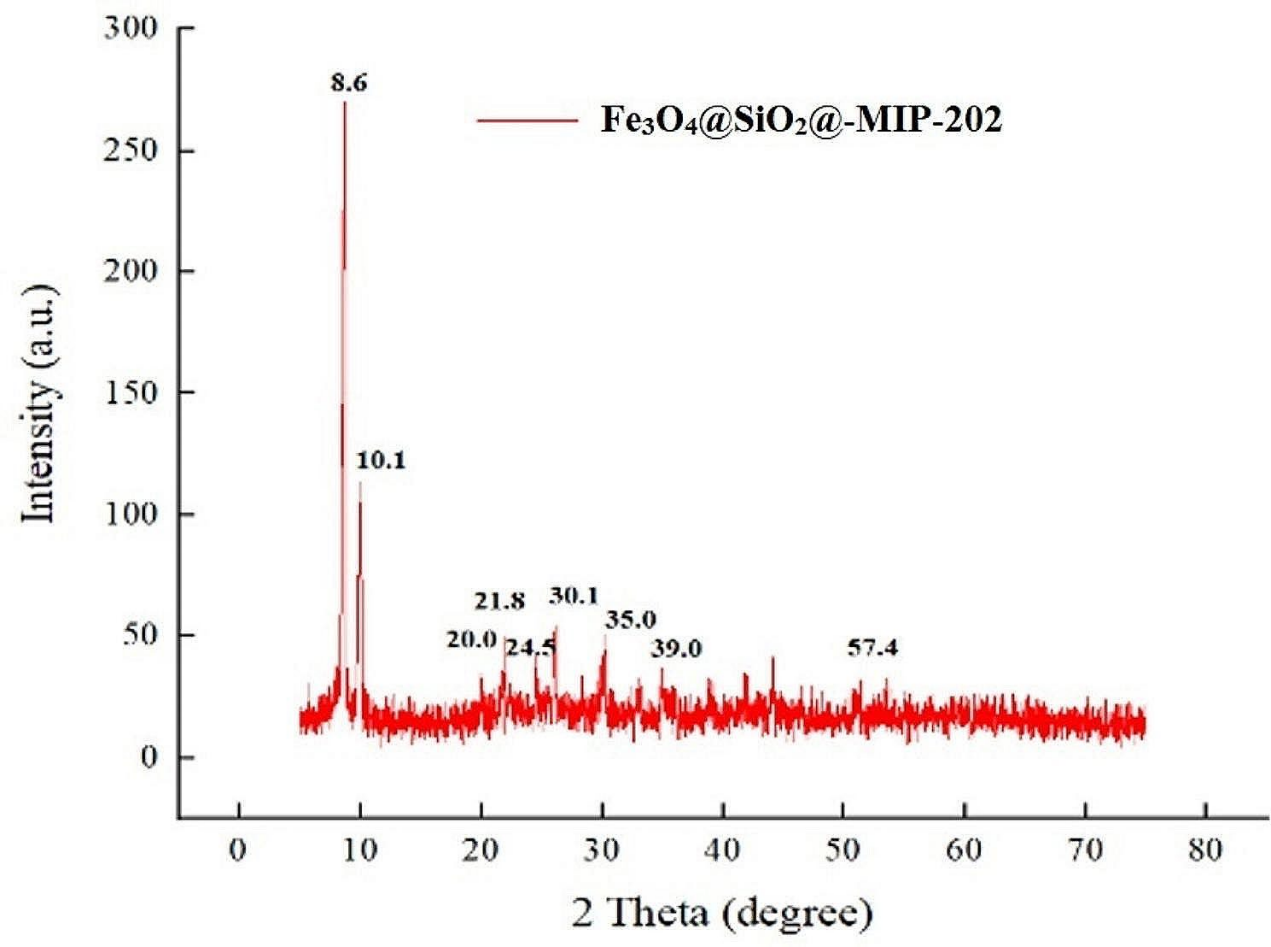
PXRD spectra of as-synthesized Fe_3_O_4_@SiO_2_@MIP-202 nanocomposite

PXRD data revealed the crystalline structure of the prepared nanocomposite (Fig. 3). Because of the presence of peaks at 30.1° (220), 35.0° (311), 39.0° (400), and 57.5° (440), an inverse cubic spinel can be suggested for Fe_3_O_4_ structure [41]. The amorphous silica shell on the surface of the nanoparticles in Fe_3_O_4_@SiO_2_ accounts for the large peaks at around 24.5° [37]. Therefore, it is proven that Fe_3_O_4_ and SiO_2_ are combined together. MIP-202 exhibited distinct maximum intensities at 8.6°, 10.1°, 20.0°, and 21.8°, which matched with the (111), (200), (420), and (440) planes, respectively. For the crystalline MIP-202 structure, these maximum intensities represent the most conspicuous and defining diffraction signals [42]. The crystal structure of MIP-202 is also very stable after formation. This also implies that the synthesized nanocomposite is a promising option for use in water treatment and may pave the way for further exploration of mixed matrix membranes based on MOFs.

**Fig. 4:**
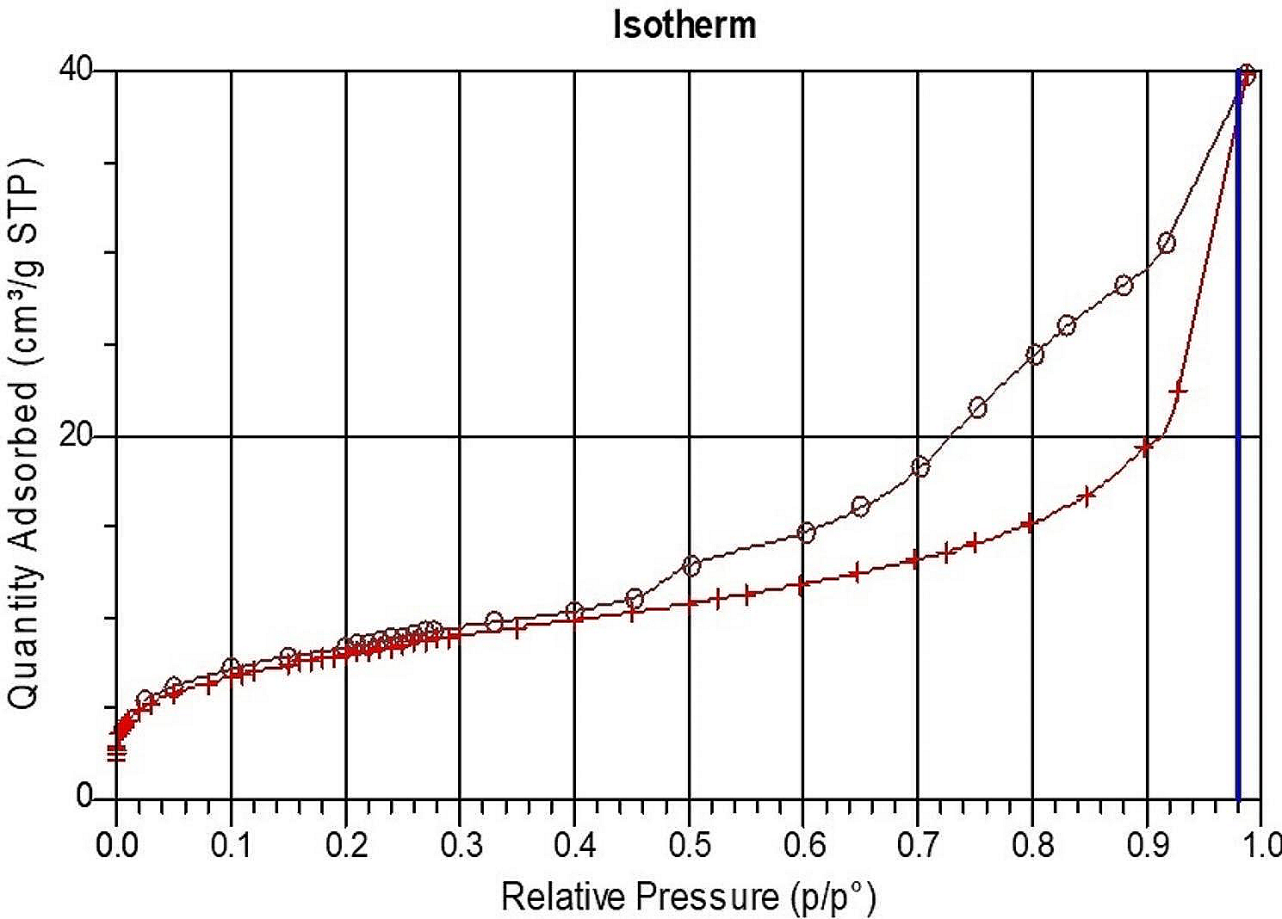
BET isotherm plots of Fe_3_O_4_@SiO_2_@MIP-202

**Fig. 5:**
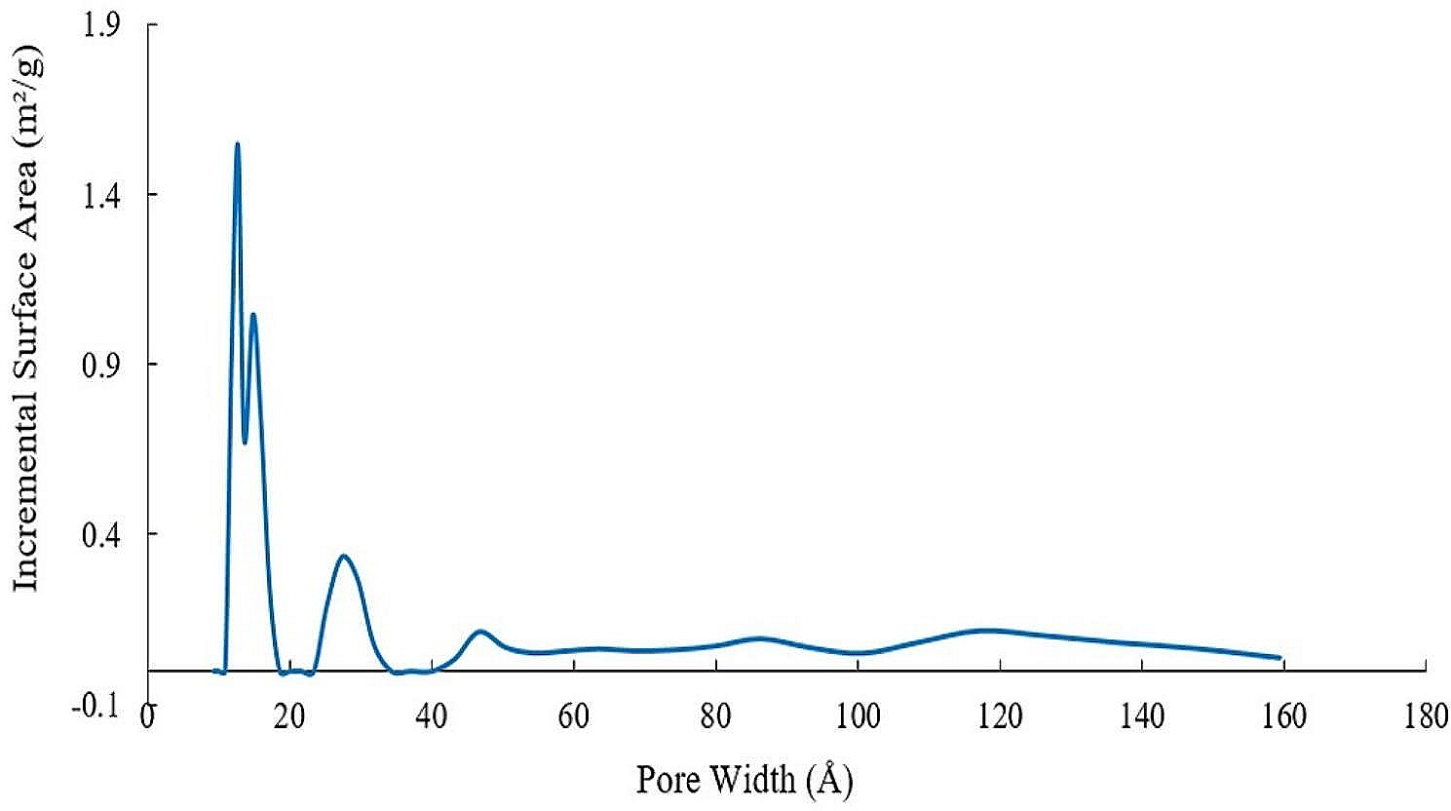
Incremental pore volume versus pore diameter recorded for Fe_3_O_4_@SiO_2_@MIP-202 nanocomposite

BET was used for the determination of surface area of the nanocomposite utilizing N_2_ isotherms (Fig. 4). The specific surface area of the MMOF was estimated at 65 m^2^.g^− 1^ with a total pore volume of 0.059 cm^3^.g^− 1^. By using BET, it was found that the surface area of the synthesized nanocomposite is higher than that of MIP-202 MMOF which indicates the improvement of the surface area of the nanocomposite absorbent after its loading on Fe_3_O_4_@SiO_2_ nanoparticles (Figs. 4 and 5). The acceptable surface area and small size of the Fe_3_O_4_@SiO_2_@-MIP-202 nanocomposite may be also due to the preparation method [43].

Thermogravimetric analysis (TGA) in nitrogen was applied for the determination of the thermal stability of the MMOF adsorbent (Fig. SI1). Between 25 and 820 °C, three distinct phases of weight loss were observed in the TGA curves of both MIP202 and Fe_3_O_4_@SiO_2_@MIP-202. Adsorbed water is evaporated at low temperatures, whereas ligands are broken down and zirconium oxide is formed at higher temperatures. Evaporation of water molecules is the primary source of the first loss in weight (4%) at temperatures below 120 °C. Water, residual solvent, and trapped ambient gases are released from the interior pores, leading to about 17% weight loss between 120 and 280 °C. High thermal stability was observed for both MIP202 and Fe_3_O_4_@SiO_2_@MIP-202 up to 280 °C. At temperatures between 280 and 475 °C, MIP202 changes into ZrO_2_ after gradually losing weight (∼46%) owing to the amine cleavage of the ligand and the disintegration of the framework structure [44]. These TGA experiments illustrate that the nanocomposite is more thermally stable than MIP-202. For the phase transition of MIP202 and Fe_3_O_4_@SiO_2_@MIP-202, differential thermal analysis (DTA) was employed (Fig. SI2). Water, solvents, and trapped gases on the adsorption sites of the surface of the Fe_3_O_4_@SiO_2_@MIP-202 structure are responsible for the first, second, and the third peaks which were observed between 32 and 163 °C, 163 and 247 °C, and 247 and 294 °C, respectively. The endothermic impact of the water discharge on the DTA curve was reached at its lowest at roughly 120 °C. The breakdown and oxidation of L-aspartic acid, together with the generation and release of ZrO_2_ and CO_2_ from MIP-202 are associated with the end peak recognized in the 294–815 °C temperature range.

**Fig. 6:**
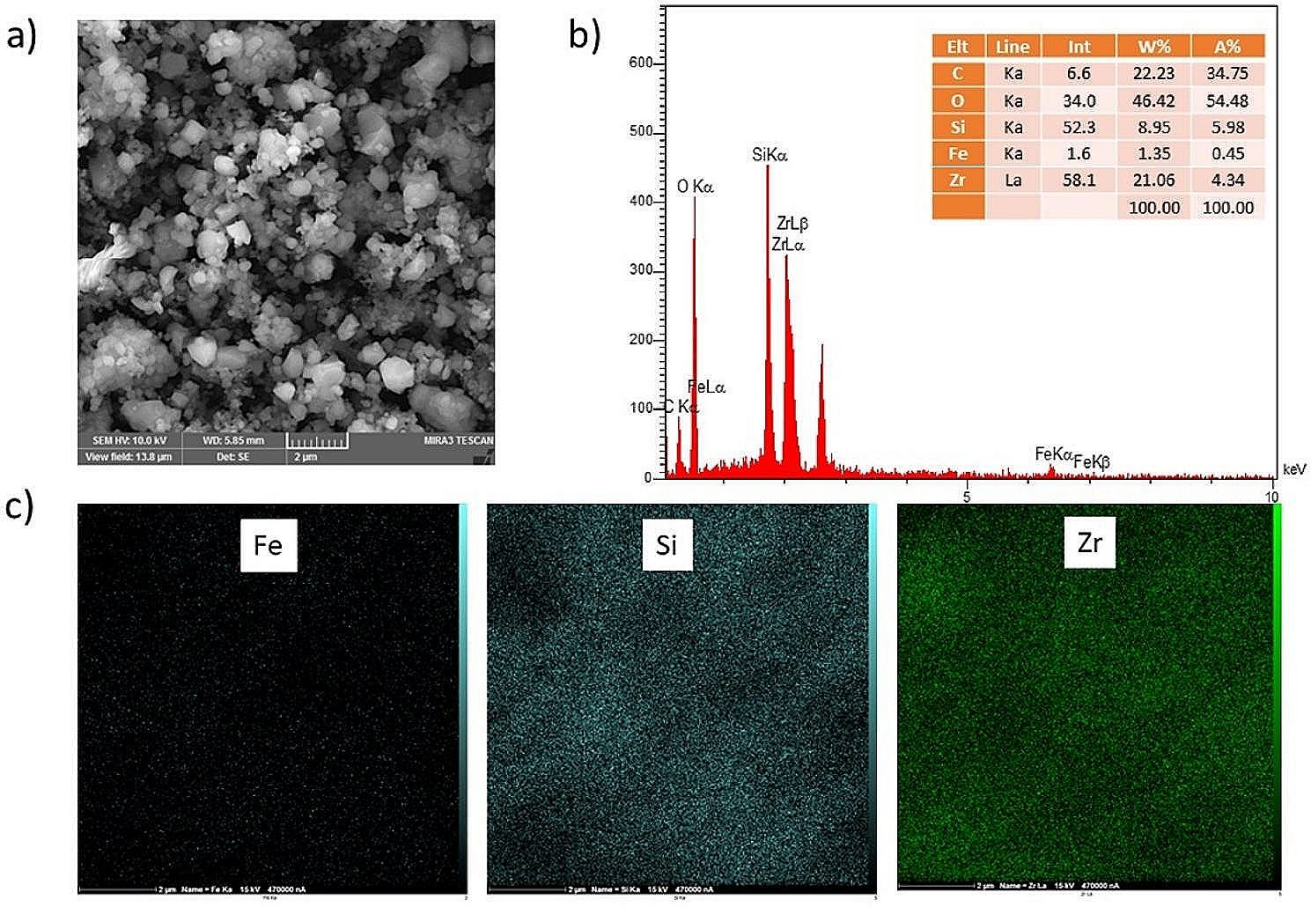
SEM image (**a**), elemental analysis (**b**) and EDX mapping (**c**) of Fe, Si and Zr for Fe_3_O_4_@SiO_2_@MIP-202

SEM images of Fe_3_O_4_@SiO_2_@MIP-202 indicated the presence of crystalline forms with a diameter of 2 μm (Fig. 6). An EDX elemental analysis of the synthesized MMOF confirmed the existence of Fe_3_O_4_, SiO_2_, and MIP-202, with representing Si, Fe, and Zr as the most abundant atoms. A table of quantitative data of the elements is presented in Fig. 6b. Nitrogen could not be detected by EDX due to its low molecular weight and low concentration as confirmed by the literature.

Employing the vibrating sample magnetometer (VSM) analysis revealed that the saturation magnetization of Fe_3_O_4_@SiO_2_@MIP-202 nanocomposites is 1.7 emu.g^− 1^ (Fig. SI3). Saturation magnetizations of Fe_3_O_4_ and Fe_3_O_4_@SiO_2_, respectively, are reported in the literature to be 67.2 and 21.5 emu.g^− 1^ [45], indicating that a thick layer of MIP-202 has been formed over Fe_3_O_4_@SiO_2_, decreasing the magnetic content of the nanocomposite. The data imply that the synthesized nanocomposite can be a promising option for use it in water treatment.

### Optimization of the extraction

Multiple variables that might have an impact on SPE extraction of Pd^2+^ were tested utilizing one-variable-at-a-time and response surface methodology (RSM). A 1000.0 mg.L^− 1^ aliquots of standard solution of Pd^2+^ was utilized in aliquots for the optimization experiments. Each experiment was performed at least in triplicate.

### Response surface methodology

To determine the optimal experimental parameters for SPE of Pd^2+^ employing RSM, an optimization procedure was conducted. RSM is a methodology for experimental design that aims to find the maximum extraction efficiency (EE). it facilitates the exploration of intricate interactions and the identification of optimal operating conditions. Furthermore, RSM can result in cost savings and heightened efficiency in research [46]. The amount of sorbent (mg) (A or X1), pH (B or X2), and eluent volume (C or X3) were presumed to be relevant factors in this study. The low, middle, and high levels of each parameter were shown as − 1, 0, and + 1, respectively (Table 1 and Tables SI1SI1-SI3). Residual versus run number and correlation of experimental versus predicted values are presented in Fig. SI4. Equation (1) approximates the mathematical relationship between the three significant system variables A (X1), B (X2), and C (X3) by utilizing a quadratic (second degree) polynomial:

**Table 1:** Designing media in the RSM model for optimization of Pd^2+^ extraction

Run order	Actual Value	Predicted Value	Residual	Leverage	Internally Studentized Residuals	Externally Studentized Residuals	Cook’s Distance	Influence on Fitted Value DFFITS	Standard Order
1	0.5330	0.5121	0.0209	0.750	2.262	4.040	1.535^(1)^	6.997^(1)^	9
2	0.4470	0.4368	0.0102	0.200	0.618	0.588	0.010	0.294	13
3	0.9820	0.9934	-0.0114	0.750	-1.234	-1.292	0.457	-2.237	6
4	0.3960	0.3899	0.0061	0.750	0.665	0.636	0.133	1.102	8
5	0.4330	0.4368	-0.0038	0.200	-0.230	-0.214	0.001	-0.107	16
6	0.1940	0.2087	-0.0147	0.750	-1.597	-1.855	0.765	-3.213^(1)^	1
7	0.2060	0.1946	0.0114	0.750	1.234	1.292	0.457	2.237	7
8	0.3140	0.3349	-0.0209	0.750	-2.262	-4.040	1.535^(1)^	-6.997^(1)^	12
9	0.4190	0.4368	-0.0178	0.200	-1.078	-1.093	0.029	-0.547	17
10	0.7661	0.7756	-0.0095	0.750	-1.028	-1.033	0.317	-1.789	2
11	0.5570	0.5475	0.0095	0.750	1.028	1.033	0.317	1.789	3
12	0.9110	0.9144	-0.0034	0.750	-0.363	-0.339	0.040	-0.588	10
13	0.4380	0.4368	0.0012	0.200	0.073	0.067	0.000	0.034	14
14	0.4470	0.4368	0.0102	0.200	0.618	0.588	0.010	0.294	15
15	0.3470	0.3436	0.0034	0.750	0.363	0.339	0.040	0.588	11
16	0.8450	0.8303	0.0147	0.750	1.597	1.855	0.765	3.213^(1)^	4
17	0.3329	0.3390	-0.0061	0.750	-0.665	-0.636	0.133	-1.102	5

1 $$\eqalign{{\rm{Y}}\, = & {\rm{\beta }}0\, + \cr & \sum {{\rm{\beta i}}\,{\rm{Xi}}} \, + \cr & \sum {{\rm{\beta ii}}\,{\rm{Xii}}} \, + \cr & \sum {{\rm{\beta ij}}\,{\rm{Xi}}\,{\rm{Xj}}} \, + \,{\rm{e}} \cr}$$

X1, X2, and X3 are the ciphered independent variables, I is the linear influence, β_ii_ is the quadratic influence, β_ij_ proves the coefficient of the interaction factor, and Y is the predicted response. In this formula, e is the random error or permits descriptions of uncertainties between predicted and achieved data [47]. Equation 2 illustrates the mathematical relationship between the analytical signal and the four provided variables.

2 $$\eqalign{{\rm{R1}}\,{\rm{ = }} & \left( {{\rm{ + 0}}{\rm{.4368}}} \right)\,{\rm{ + }}\,\left( {{\rm{0}}{\rm{.2124}}\,{\rm{ \times }}\,{\rm{A}}} \right){\rm{ + }} \cr & \left( {{\rm{0}}{\rm{.0984}}\,{\rm{ \times }}\,{\rm{B}}} \right)\, - \,\left( {{\rm{0}}{\rm{.1870}}\,{\rm{ \times }}\,{\rm{C}}} \right)\, - \cr & \left( {{\rm{0}}{\rm{.0710}}\,{\rm{ \times }}\,{\rm{AB}}} \right)\, - \,\left( {{\rm{0}}{\rm{.1148}}\,{\rm{ \times }}\,{\rm{AC}}} \right) - \cr & \left( {{\rm{0}}{\rm{.1027}}\,{\rm{ \times BC}}} \right){\rm{ + }}\left( {{\rm{0}}{\rm{.0533}}\,{\rm{ \times }}\,{{\rm{A}}^{\rm{2}}}} \right){\rm{ + }} \cr & \left( {{\rm{0}}{\rm{.1004}}\,{\rm{ \times }}\,{{\rm{B}}^{\rm{2}}}} \right)\, - \,\left( {{\rm{0}}{\rm{.0109}}\,{\rm{ \times }}\,{{\rm{C}}^{\rm{2}}}} \right) \cr}$$

**Fig. 7:**
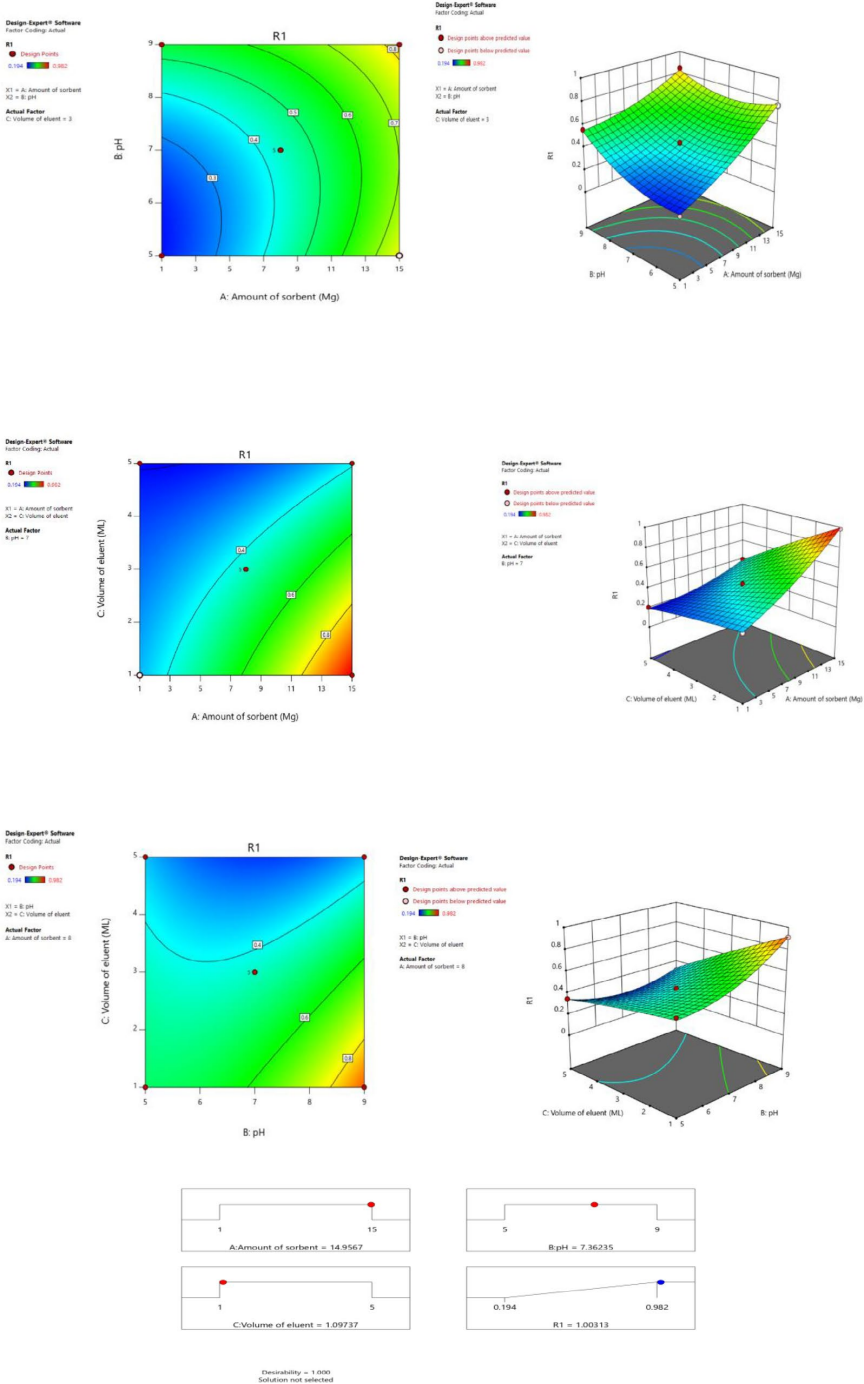
Response surface plot (3D) and contour plot (2D) showing the effects of variables (pH, amount of sorbent and volume of eluent) on solid phase extraction of Pd^2+^ ions (response) and desirability of the used approach

The critical point on the surface may be found by solving these systems of equations under the conditions ∂(Y)/∂(A) = 0, ∂(Y)/∂(B) = 0 and ∂(Y)/∂(C) = 0 [48]. Here are the attained crucial paints: a sorbent amount (A) of 15 mg, pH of 7.4, and eluent volume (C) of 1 mL. The isoelectric point of the Fe_3_O_4_@SiO_2_@-MIP-202 adsorbent was determined to be pH 7.15. Above this pH, the surface charge of the adsorbent becomes net negative, while below pH 7.15 the net surface charge is positive. Under conditions at pH 7.4, greater than the measured isoelectric point, the Fe_3_O_4_@SiO_2_@-MIP-202 adsorbent surface takes on a net negative charge. Since the Pd^2+^ ions targeted for adsorption carry a positive charge (cations), there is an increased electrostatic attraction between the negatively charged adsorbent surface and the positively charged Pd^2+^ at pH 7.4. This electrostatic interaction enhances the contact and binding of the Pd^2+^ ions to the Fe_3_O_4_@SiO_2_@-MIP-202 adsorbent, thereby improving the recovery and uptake of palladium from solution under these conditions. The determination coefficient (R^2^ = 0.9993) for the analyte regression model explains why this model can account for all but 0.23% of the variance. This model was exhibited to be statistically significant with an adjusted R^2^ = 0.9939. The resultant prediction is also highly effective, with an R^2^ of 0.9260, which is an unfavorable degree of agreement. An F-value demonstrates model relevance (Tables SI2 and SI3). A p-value of less than 0.0001 indicated that regression models were highly significant. If the p-value in the ANOVA table is less than 0.05, then the influence is statistically significant at the 95% confidence level. The parameter was not statistically significant when compared to the pure errors, as displayed by the F-value of lack of fit which was 4.54. The ANOVA of the model was very encouraging due to the small discrepancy between the anticipated data and the experimental outcome. Utilizing an extremely small probability value (p_model_ ≤ 0.0001), the quadratic model was significant, according to an ANOVA of the regression model. The effect of the independent variables on the response (extraction of Pd2+) is described by employing 2D and 3D diagrams (Fig. 7). Interactions of influencing factors (pH and amount of sorbent), (volume of eluent and amount of sorbent) and (volume of eluent and pH) on solid phase extraction of Pd^2+^ ions were investigated and the results are depicted in Fig. SI5.

### Analytical figures of merit

Under the optimum extraction conditions, the analytical performance of the developed method was evaluated. The linearity of the method was examined utilizing standard solutions by increasing the concentrations of Pd^2+^. The calibration curve (absorbance vs. concentration) indicated a linear range across the 10.0 to 1500.0 mg.L^− 1^, by equation and determination coefficients (R^2^) of A = 0.0009 C (µg.L^− 1^) + 0.139 and 0.998, respectively. The limit of detection (LOD) was obtained as 1.05 µg.L^− 1^ using 3(S_d_)/m criteria [49], in which S_d_ is the standard deviation of the blank determination for 10 experiments and *m* is the slope of the calibration curve. Relative standard deviation (RSD%, *n* = 5, C = 0.1 mg.L^− 1^) was applied to characterize the accuracy on the same day The repeatability of the measurements was found to be 1.8%. To calculate the adsorption capacity of the Fe_3_O_4_@SiO_2_@-MIP-202, a 1000 mg.L^− 1^ solution of Pd^2+^ was extracted with 15 mg of the adsorbent with continuous stirring for 10 min. The adsorption capacity was obtained as 194.52 mg of Pd^2+^ ions/g of the adsorbent based on the equation *q*=(*C*_*i*_–*C*_e_)*V*/*m*, where *q* is the maximum adsorption capacity (mg/g), *C*_i_ is the initial concentration of the standard solution (mg/L), *C*_e_ is the concentration after extraction (mg/L), *V* is the volume of the sample solution (mL) and *m* is the mass of adsorbent (g). The adsorbent demonstrated the ability to be regenerated and reused for at least five cycles without a significant change in its capacity. These findings demonstrated the reliability and consistency of the technique for the extraction of Pd^2+^ from aqueous samples. The results of this work is close to a study where pyridyltriazole-functionalized UiO-66 (UiO-66-Pyta) served as an adsorbent for extracting palladium ions [50]. The authors showed that UiO-66-Pyta is a selective adsorbent towards Pd^2+^ with an adsorption capacity of 294.1 mg.g^− 1^ at an acidic pH (4.5). This article confirms that MOF-based adsorbents possess the capability to extract palladium ions with an efficiency of over 95%. In order to calculate the extraction efficiency, the EE%=100(C_B_/C_A_) relation was used [50], in which C_A_ and C_B_ are the concentrations of Pd^2+^ in the solution before and after extraction, respectively. EE% calculated by the mentioned relation was 98%. Knowing the value of the EE, the enrichment factor (EF) was calculated by dividing the sample volume (V_i_, 250.0 mL) by the eluent volume (V_e_, 1.0 mL), and a value of 245 was obtained for it. The total analysis time, including extraction and spectrophotometric determination was less than 30 min. Table 2 compares the characteristic data of the present method to those recently reported in the literature for the same analyte. The results showed that the LOD in the study (1.05 µg.L^− 1^) is better than what obtained in ref [50]. (1.9 µg.L^− 1^) and ref [51]. (90 µg.L^− 1^). However, the LOD values of refs [52] and [53]. are 0.0012 µg.L^− 1^ and 0.12 µg.L^− 1^, respectively, which are better than our LOD value. This shows that LOD obtained in this work compared to those reported in the literature is acceptable and somewhat weak. In addition, the linear concentration range obtained in this work is wider compared to the reported values in the literature, which is one of its advantages. In addition, the introduced nanocomposite has magnetic properties and can be easily removed from the sample solution.

**Table 2:** Comparison of the figures of merit of the developed methods with different sorbents reported in the literature

Matrix	Extraction method/Instrument used	Adsorbent	Adsorption capacity (mg of Pd^2+^ ions/g of the adsorbent)	EF	LOD (µg/L)	Linear range (µg/L)	RSD (%)	Ref.
Water	SPE/UV^1^	UiO-66-Pyta^2^	294.1	NM^3^	1.9	NM	1.7	[50]
Sulfide ores	SPE/FIA-FAAS^4^	SSPS^5^	NM	NM	5.0	NM	0.7	[51]
Road dust	MSPE/FAAS^6^	MGOSDN^7^	41.4	250	0.0012	0.003-2.5	2.2	[52]
Soil	D-µ-MSPE/FAAS^8^	MN-SDS/5-Br-PDA^9^	NM	NM	0.12	NM	1.8	[53]
Water and wastewater	SPE/UV	Fe_3_O_4_@SiO_2_@-MIP-202	194.52	245	1.05	10.00-1500.00	1.8	This work

^1^SPE/UV: Solid-phase extraction/spectrophotometer; ^2^Pyta: Pyridyltriazol; ^3^NM: not mentioned; ^4^SPE/FIA-FAAS: Solid-phase extraction/flow injection analysis-flame atomic absorption spectrometry; ^5^SSPS: 4-(n-octyl)diethylenetriamine and hyper cross-linked polystyrene; ^6^MSPE/FAAS: Magnetic solid phase extraction/flame atomic absorption spectrometry; ^7^MGOSDN: Magnetic graphene oxide silicon dioxide nanocomposite; ^8^D-µ-MSPE/FAAS: Dispersive micro magnetic solid phase extraction/flame atomic absorption spectrometry; ^9^MN-SDS/5-Br-PDA: Magnetic nanoparticles coated by sodium dodecyl sulfate and 2-(5-bromo-2-pyridylazo)-5-diethyl aminophenol ligand

### Effect of interfering ions

To be a suitable technique for extraction, the suggested method should be able to have a high selectivity towards Pd^2+^ and extract it with high efficiency from the sample solution in the presence of similar ions. To investigate the selectivity of the adsorbent toward Pd^2+^ a 1.0 mg.L^− 1^ standard solution of it was prepared, and foreign ions that may potentially be present in a typical wastewater sample (as listed in Table 3) were added separately to make a “two-component” system. The greatest concentration of an interfering ion that yields an EE error of less than ± 5% is referred to as the tolerance limit. Most of the other cations of transition elements such as nickel and cadmium showed no interference at any concentrations. According to Table 3, Pd^2+^ can be selectively extracted in the presence of interfering ions employing the Fe_3_O_4_@SiO_2_@MIP-202 adsorbent, and the existence of these ions even at high concentrations does not affect the preconcentration and separation of Pd^2+^. This preference arises from Pd^2+^ comparatively smaller ionic radius (86 pm) and higher charge density when compared to the other metallic cations. Furthermore, Pd^2+^ falls into the category of soft acids, differing from hard acids like Mg^2+^ or Ca^2+^. As a result, it exhibits a preference for binding with nitrogen soft bases found in the Zr-MOFs, and tends to adopt a square planar configuration [54].

**Table 3:** Interfering ions and their effect on Pd^2+^ detection limit

Interfering ion	Tolerance limit (mg.L^− 1^)
Li^+^, Na^+^, K^+^	100
Ca^2+^, Mg^2+^, Ba^2+^	100
Ag^+^, Zn^2+^, Hg^2+^, Co^2+^, Mn^2+^, Cu^2+^, Fe^2+^, Pb^2+^, Sn^2+^, Cr^3+^	10

### Effect of extraction time

Contact time between the extracting solid phase and the sample solution is an important parameter in SPE that should be carefully optimized. Extraction times between 5.0 and 25.0 min were examined for a standard solution containing 1000 µg.L^− 1^ Pb^2+^ ions (Fig. SI6). EE was increased sharply up to 5.0 min, after that, it was increased slowly up to 10 min, and then became constant. As a result, 10.0 min was chosen as the best time for extraction. This is because after 5 min, the adsorption sites of the nanocomposite are partially saturated with Pd^2+^ ions, and after 10 min they are completely occupied, so after 10 min, no more Pd^2+^ ions can be adsorbed on the adsorbent surface.

### Effect of desorption time

The desorption period of Pd^2+^ from the adsorbent is another crucial parameter that should be optimized. Interaction between the adsorbate and the adsorbent has a direct influence on the time of desorption of analytes from the adsorbent. Desorption time was varied between 5.0 and 20.0 min (Fig. SI7) and it was observed that the best EE occurred for 15.0 min desorption of Pd^2+^ from the MMOF by the eluting solvent (1.0 mL HNO_3_). As a result, this time was chosen as ideal time for Pd^2+^ desorption and full elution.

### Reusability of the adsorbent

The ability of the MMOF adsorbent to be reused for Pd^2+^ extraction was examined. After five successive extractions, EE was dropped to 84.5%. This is probably because some of the Pd^2+^ ions may be tightly bonded to the amine and carboxyl groups of MMOF, which prevents the incomplete desorption of the Pd^2+^ ion and causes a slight decrease in the overall active sites available for the next extraction. As a result, the adsorbent was changed after performing five extractions.

### Effect of sample volume

Since the sensitivity of SPE method is proportional to the amount of the analyte presents in the sample, it needs to be optimized. Increasing the sample volume is expected to decrease the responses. However, if the sample volume significantly exceeds the adsorption capacity of the adsorbent, increasing the sample volume cannot change the response. In this research, the effect of sample volume between 25.0 mL to 500.0 mL was investigated, while each solution containing 1.0 mg.L^− 1^ of Pd^2+^. It was found that the maximum EE (98%) occurs at 250.0 mL.

### Real sample analysis

To investigate the effect of sample media on analytical signal and feasibility of the developed method for real samples analysis, samples were taken from local groundwater, well water and wastewater (municipal sewage). Filter paper was utilized to remove any suspended particles from wastewater before its analysis. No analyte was found in them. To investigate the matrix effect on the extraction, samples were spiked at three levels of 100, 500, and 1200 µg.L^− 1^ with Pd^2+^ (Table 4) and extraction was performed at the optimum conditions. Excellent recoveries (95.2–96.8%) were observed. Moreover, repeatability (RSD%) was better than 2.1% which demonstrates that the adsorbent has good efficiency for the extraction of Pd^2+^ from complicated matrices. For validation of the method, a groundwater sample was selected and spiked to have a concentration of 100 µg.L^− 1^ of Pd^2+^ and analyzed with the developed method and a concentration of 96.5 µg.L^− 1^ with an RSD% of 1.6% (*n* = 3) was obtained. The same spiked sample was analyzed with a graphite furnace atomic absorption spectrometer, according to the EPA standard method 253.2 [55]. Triplicate analysis, resulted a concentration of 98 µg.L^− 1^ with an RSD of 1.3%. Considering the lack of significant difference between the obtained results between the developed and the standard method, the suggested method can be considered to be valid.

**Table 4:** Real samples analysis results

Sample	Pd^2+^ (µg.L^− 1^)		Recovery (%)	RSD% (*n* = 3)
	Added	Found		
Ground water	0	0	---	0
	100	96.5	96.5	1.6
	500	484	96.8	1.7
	1200	1160.4	96.7	1.4
Well water	0	0	---	0
	100	95.7	95.7	1.8
	500	479	95.8	2.1
	1200	1146	95.5	2.4
Wastewater	0	0	---	0
	100	95.3	95.3	1.9
	500	476	95.2	1.5
	1200	1149.6	95.8	1.7

## Conclusion

In this work, a magnetically bio-based Zr-MOF (Fe_3_O_4_@SiO_2_@MIP-202) was synthesized and employed as an adsorbent for selective extraction of Pd^2+^ from aqueous media. FT-IR, PXRD, SEM/EDX, BET, VSM, and TGA were applied to characterize structural and morphological features of this MMOF. The synthesized MMOF showed a high adsorption capacity because of the presence of numerous free active Zr sites and hydroxyl groups, with high porosity, and large surface area. Analytical performance evaluation demonstrated a wide linear response between 10.00 and 1500.00 µg.L^− 1^, and a remarkably low LOD (1.05 µg.L^− 1^). EE and EF were calculated as 98.0% and 245, respectively. The experiments showed that Fe_3_O_4_@SiO_2_@-MIP-202 can act as a selective adsorbent towards Pd^2+^ ions. No special sample pre-treatment was required before the extraction and there is no need to use toxic organic solvents. The adsorbent could be used for at least five extractions without substantial change in its adsorption power, with the total analysis time was less than 30 min. Analysis of spiked samples of groundwater, well water, and municipal wastewater exhibited good extraction efficiencies (95.2–96.8%) and repeatability (RSD < 2.1%). The other advantages of this method are the use of the conventional spectrophotometer and easy magnetically separable Zr-MOF, and no need to sample pre-treatment and organic solvents.

### Electronic supplementary material

Below is the link to the electronic supplementary material.

Supplementary Material 1

## Data Availability

The majority of the data used to support the findings of this study are included within the article and the on-line Supplementary Information. Other data are available from the corresponding author upon request.

## Abbreviations

BBD: Box-Behnken Design
DTA: Differential Thermal Analysis
EE: Extraction Efficiency
EF: Enrichment Factor
LOD: Limit of Detection
MOF: Metal Organic Framework
RSM: Response Surface Methodology
SPE: Solid Phase Extraction
TGA: Thermogravimetric Analysis
VSM: Vibrating Sample Magnetometer
XRD: X-ray Diffraction
