# Supplementary material for: Application of a magnetically separable Zr-MOF for fast extraction of palladium before its spectrophotometric detection
Source: BMC Chem. 2024 Mar 30;18(1):63. doi: 10.1186/s13065-024-01171-w (PMC10981821; doi:10.1186/s13065-024-01171-w)
Supplement: Supplementary file 1 — Supplementary Material 1 [file 13065_2024_1171_MOESM1_ESM.docx]

**Supplementary data to:**

**Application of a magnetically separable Zr-MOF for fast extraction of palladium before its spectrophotometric detection**

*Amin Piri^1^, Massoud Kaykhaii^[[1]](#footnote-1),*^, Mostafa Khajeh^2^ and Alireza Oveisi^2^*

^1^Department of Chemistry, Faculty of Sciences, University of Sistan and Baluchestan, Zahedan 98135-674, Iran

^2^Department of Chemistry, University of Zabol, P.O. Box: 98615-538, Zabol, Iran

###
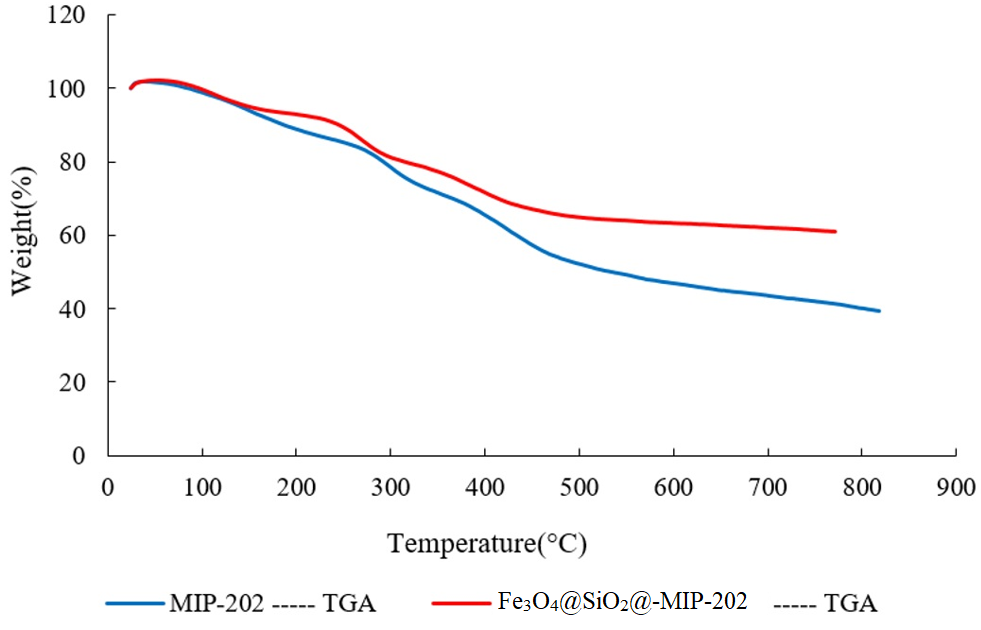


**Fig. SI1.** TGA of MIP-202 and Fe_3_O_4_@SiO_2_@-MIP-202 nanocomposite.

###
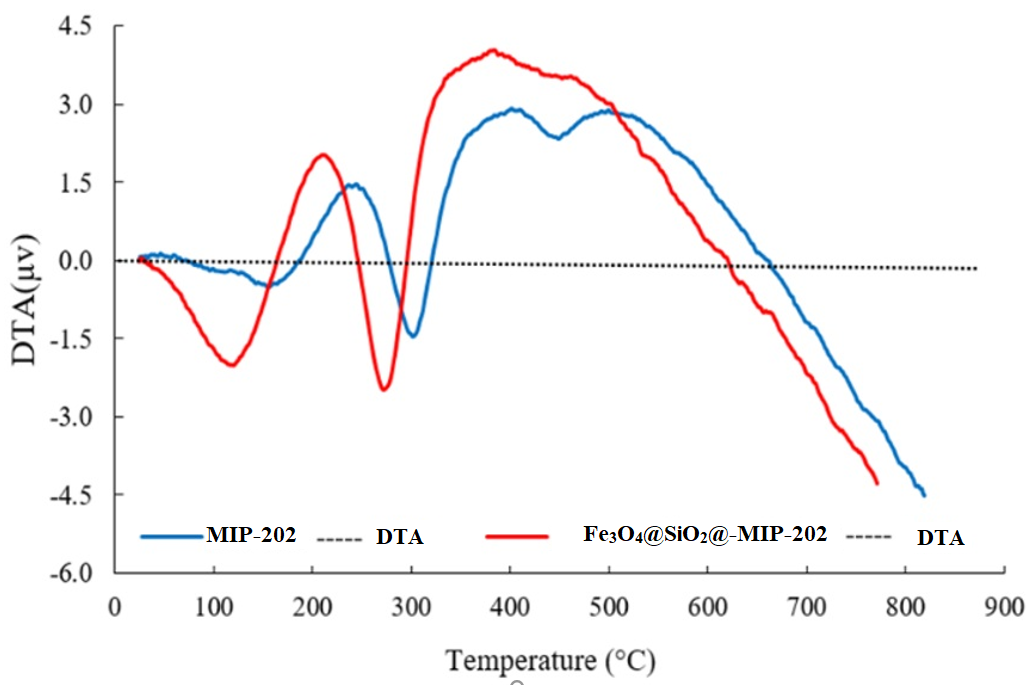


**Fig. SI2.** DTA curves of MIP202 and Fe_3_O_4_@SiO_2_@-MIP-202 nanocomposite.

**
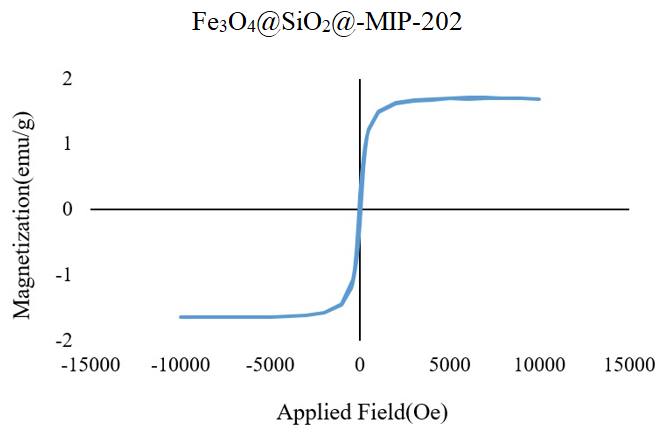
**

**Fig. SI3.** VSM analysis of Fe_3_O_4_@SiO_2_@-MIP-202.

|  | 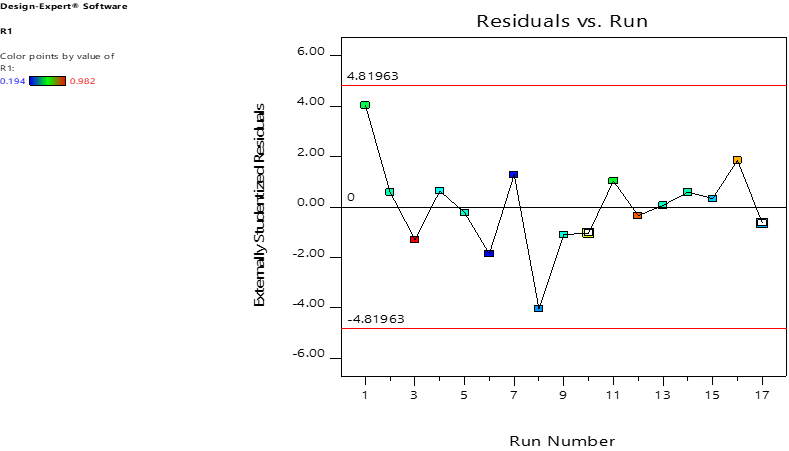 |
| --- | --- |

###
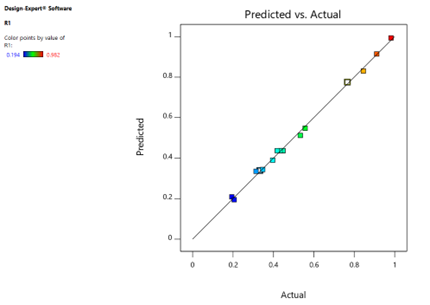


(b)

(a)

### Fig. SI4. Residual versus run number using BBD method (a), correlation of experimental versus predicted values (b)


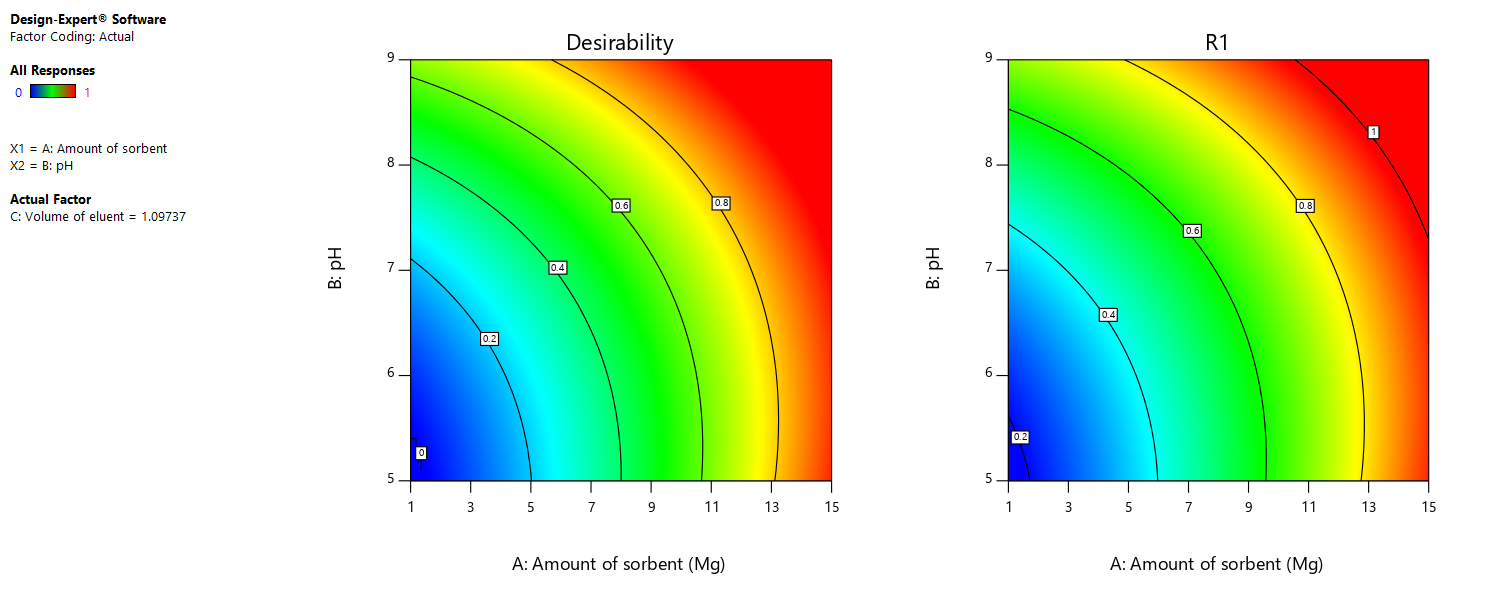


(a)


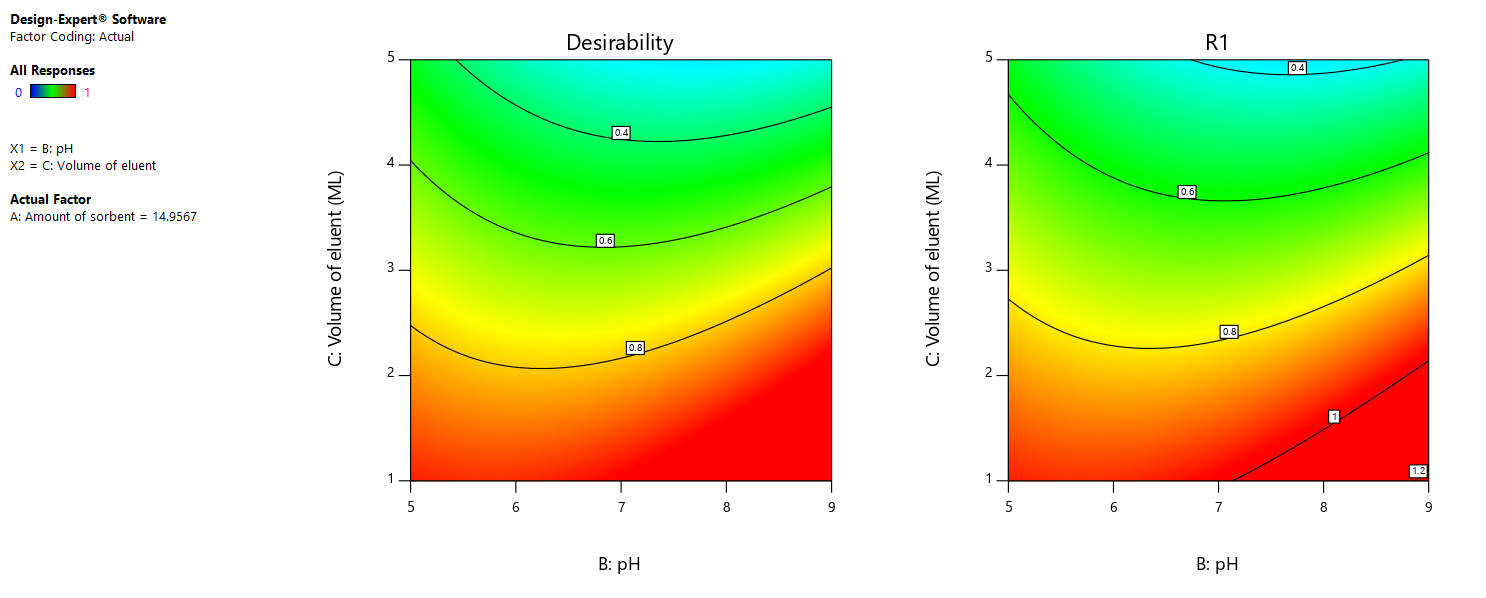


(b)


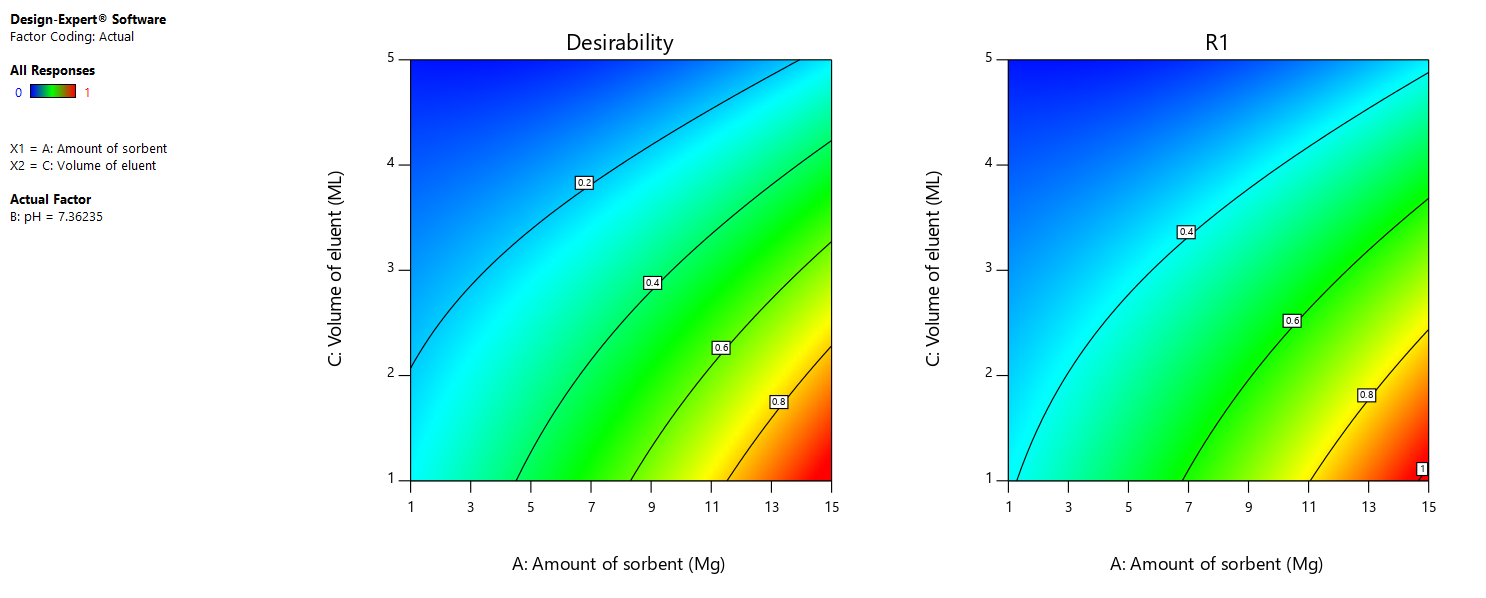


(c)

**Fig. SI5.** Interactions of influencing factors pH and amount of sorbent **(a)**, volume of eluent and amount of sorbent **(b)**, volume of eluent and pH on solid phase extraction of Pd^2+^ ions **(c)**

###
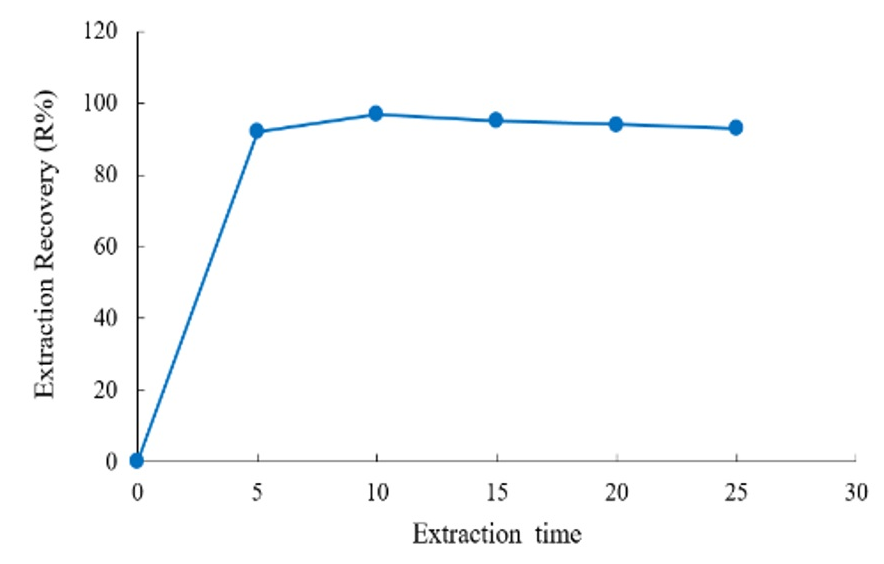


**Fig. SI6.** The effect of extraction time on extraction recovery.

###
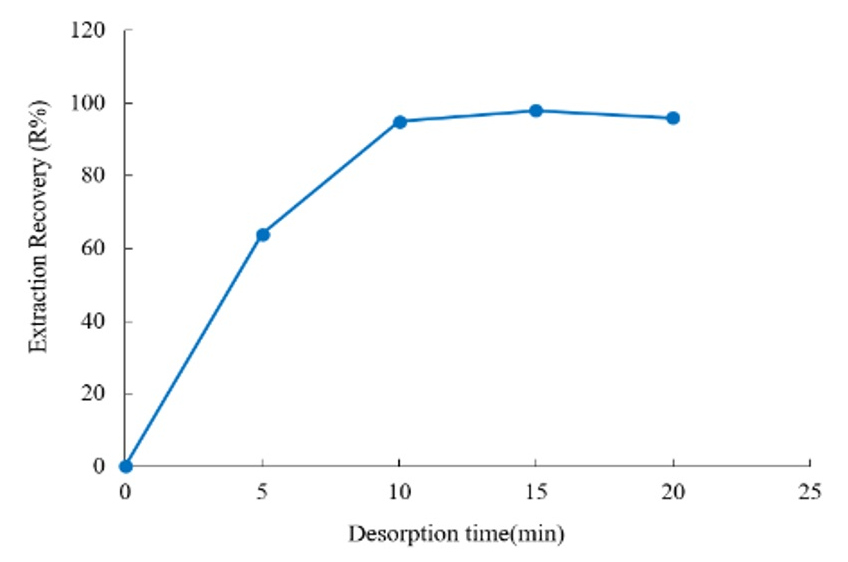


**Fig. SI7.** The effect of desorption time on extraction recovery.

### Table SI1. Design matrix for Pd^2+^ extraction.

|  | Factor 1 | Factor 2 | Factor 3 | Response 1 |
| --- | --- | --- | --- | --- |
| Run | A:Amount of sorbent (mg) | B:pH | C:Volume of eluent (mL) | Absorbance |
| 1 | 8 | 5 | 1 | 0.533 |
| 2 | 8 | 7 | 3 | 0.447 |
| 3 | 15 | 7 | 1 | 0.982 |
| 4 | 15 | 7 | 5 | 0.396 |
| 5 | 8 | 7 | 3 | 0.433 |
| 6 | 1 | 5 | 3 | 0.194 |
| 7 | 1 | 7 | 5 | 0.206 |
| 8 | 8 | 9 | 5 | 0.314 |
| 9 | 8 | 7 | 3 | 0.419 |
| *10* | *15* | *5* | *3* | *0.7661* |
| 11 | 1 | 9 | 3 | 0.557 |
| 12 | 8 | 9 | 1 | 0.911 |
| 13 | 8 | 7 | 3 | 0.438 |
| 14 | 8 | 7 | 3 | 0.447 |
| 15 | 8 | 5 | 5 | 0.347 |
| 16 | 15 | 9 | 3 | 0.845 |
| *17* | *1* | *7* | *1* | *0.3329* |

### Table SI2. Analysis of variance for Pd^2+^ extraction.

| **Source** | **Sum of Square** | **df** | **Mean Square** | **F-value** | **p-value** |  |
| --- | --- | --- | --- | --- | --- | --- |
| **Model** | 0.8901 | 9 | 0.0989 | 290.41 | < 0.0001 | significant |
| A-Amount of sorbent | 0.3609 | 1 | 0.3609 | 1059.77 | < 0.0001 |  |
| B-pH | 0.0774 | 1 | 0.0774 | 227.28 | < 0.0001 |  |
| C-Volume of eluent | 0.2797 | 1 | 0.2797 | 821.35 | < 0.0001 |  |
| AB | 0.0202 | 1 | 0.0202 | 59.25 | 0.0001 |  |
| AC | 0.0527 | 1 | 0.0527 | 154.73 | < 0.0001 |  |
| BC | 0.0422 | 1 | 0.0422 | 124.00 | < 0.0001 |  |
| A^2^ | 0.0120 | 1 | 0.0120 | 35.19 | 0.0006 |  |
| B^2^ | 0.0424 | 1 | 0.0424 | 124.57 | < 0.0001 |  |
| C^2^ | 0.0005 | 1 | 0.0005 | 1.48 | 0.2638 |  |
| **Residual** | 0.0024 | 7 | 0.0003 |  |  |  |
| Lack of Fit | 0.0018 | 3 | 0.0006 | 4.54 | 0.0889 | not significant |
| Pure Error | 0.0005 | 4 | 0.0001 |  |  |  |
| **Cor Total** | 0.8925 | 16 |  |  |  |  |
| Factor coding is **Coded**. The sum of squares is **Type III - Partial** Model significance is illustrated by an F-value of 290.41. An F-value this high could only have been produced by random chance, which has a probability of just 0.01%. | | | | | | |

**Exceeds limits**

The equation defined versus actual factors may be utilized to predict the outcome for a certain level of each ingredient. In this case, it is important to use the original units when describing the amounts of each element. This equation should not be used to determine the relative importance of each component since the coefficients are scaled to match the units of each element and the intercept is not in the middle of the design space. A lack of Fit F-value of 4.54 indicates that an 8.89% probability exists that such a significant number may be attributable to random chance alone. The difference between the predicted R^2^ (0.9660) and adjusted R^2^ (0.9939) is less than 0.2, which shows the compatibility of these two values together. **With Adeq Precision, the signal/noise ratio may be evaluated. In an ideal state, this ratio must be larger than 4. In this work, the signal/noise ratio is 56.436, showing that it is rather good. Utilizing this model, you may further explore potential layout options.**

**Table SI3**. Fit summary of the RSM model for the extraction of Pd^2^.

| Std. Dev. | 0.0185 | R² | 0.9973 |
| --- | --- | --- | --- |
| Mean | 0.5040 | Adjusted R² | 0.9939 |
| C.V. % | 3.66 | Predicted R² | 0.9660 |
|  |  | Adeq Precision | 56.4360 |

### Final Equation Versus Coded Factors:

### R1= + 0.4368 + 0.2124 A + 0.0984 B - 0.1870 C - 0.0710 AB - 0.1148 AC- 0.1027 BC + 0.0533 A^2^ + 0.1004 B^2^ - 0.0109 C^2^

### For a given level of each coded component, the response may be predicted by applying the corresponding equation. By default, 1 is assigned to a high level of a factor, whereas -1 is assigned to a low level. The relative significance of the components may be calculated utilizing the encoded equation by comparing the factor coefficients.

### Final Relation versus Actual Factors:

### R1= +0.384669 + 0.073030 × Amount of sorbent - 0.184483 × pH + 0.168292× Volume of eluent -0.005073 × Amount of sorbent × pH -0.008198 × Amount of sorbent × Volume of eluent -0.025687× pH × Volume of eluent +0.001089 × Amount of sorbent² +0.025094 × pH² -0.002731 × Volume of eluent²

The relation written regarding the real factors enables one to foretell the consequence for a given level of each ingredient. Here, those same units should be employed to denote each element’s strength. It is not possible to use this equation to calculate the relative influence of each component since the coefficients are scaled to fit the units of each element and the intercept is not in the middle of the design space.

1. *Corresponding author. Tel: +98 (54) 33446413; Fax: +98 (54) 33431067; E-mail: kaykhaii@chem.usb.ac.ir [↑](#footnote-ref-1)
